# Supplementary material for: Bored to death: Artificial Intelligence research reveals the role of boredom in suicide behavior
Source: Front Psychiatry. 2024 May 3;15:1328122. doi: 10.3389/fpsyt.2024.1328122 (PMC11112344; doi:10.3389/fpsyt.2024.1328122)
Supplement: Supplementary file 1 [file DataSheet_1.pdf]

Bored to Death: Artificial Intelligence Research Reveals the Role of Boredom in Suicide Behavior

## Supplementary Material

## Primary dataset from Facebook

The complete description of the Facebook dataset used in the current study is available in our previous publication that focused on prediction of suicide risk, rather than on understanding of the suicide phenomenon.[1] However, two characteristics differentiate between the current dataset and the previous one. First, the current sample (*N* = 1,006) included four additional participants whose Facebook activity could not be investigated with the (less advanced) language model that was used in our previous study. Second, the number of Facebook postings in the current dataset (*N* = 228,052) was larger than the number of postings in the previous dataset as it included also (textual) postings that were attached to videos, images, and internet links. This is in contrast to our previous study, which focused on standalone textual postings only.

## 5-step research pipeline

As mentioned in the main article, the procedures of the study consisted of a 5-step research pipeline (Figure 1, main article). Below is a detailed description of the five steps.

### Step 1. Representing the Facebook texts

In the first step, we extracted numeric representations of the Facebook texts we collected, using a popular Deep Language Model (DLM) named Sentence-BERT (SBERT).[2] SBERT is a variant of the well-established BERT model,[3] and it is specifically suitable for generating representations of short texts, such as social media postings.

In addition to the two objectives that served the development and training of the original BERT model ("masked language model" and "next sentence prediction"),[3] SBERT has been further optimized to generate sentence representations that reflect similarities between sentences. These representations appear in the form of numerical, multidimensional vectors known as "embedding vectors", which have been demonstrated to capture many linguistic and semantic aspects of the

language.[2] In this study, we utilize the "base" version of SBERT (via the implementation of https://huggingface.co/sentence-transformers), which takes as input the entire sentence and provides a 768-dimensional vector.

These vectors are also known as 'embedding vectors' and previous studies showed that they are able to capture many linguistic and semantic aspects of natural language.[2] Altogether, the representation process resulted in 228,052 vectors, one for each Facebook text.

**Step 2. Clustering the text representations into topics**

In the second step, we applied a clustering algorithm with the goal of organizing the different posts, which were now represented numerically, into groups that ideally capture meaningful ‘topics’ (we refer to the resulted clusters as topics because the spatial proximity of the representations is assumed to reflect proximity in the semantic meaning of the posts). This is achieved by projecting posts into a numerical space with semantic significance. Consequently, posts sharing similar subjects or themes tend to belong to the same cluster, which can be labeled with the common topic represented by the posts within it. Note that there are many other traditional methods for “topic modeling” (e.g., LDA: Latent Dirichlet Allocation)[4] that are based on statistical distributions of words/n-grams in the text. However, in this study we leveraged the power of the more rich and fine-grained embedding representations that capture the entire context of the words and not just the words themselves. Furthermore, the clustering method ensures that each post is assigned to only one topic, a property that is crucial for subsequent analysis (see Step 3).

The clustering algorithm that was used for this task was HDBSCAN.[5] This algorithm applies a hierarchical density-based clustering method and automatically determines the optimal number of clusters (minimum cluster size = 25). Before applying the algorithm, the embedding representation vectors were dimensionally reduced from 768 to 10 dimensions using the Uniform Manifold Approximation and Projection (UMAP) algorithm.[5] Altogether, this process yielded 771 topics, of which we removed 60 topics that were shared by less than ten different users, in order to avoid esoteric

topics that are used only by very few. The number of posts in each topic ranged from 25 to 4956 (*M* = 96.9, *SD* =259.9 ).

**Step 3. Identifying the topics that are most related to suicide risk**

In the third step, we aimed to filter out irrelevant topics and identify the topics that are mostly correlated with suicide risk. To achieve this aim, we implemented a stepwise regression model[6] that predicts the users' suicide risk score (i.e., the CSSRS scores, which ranged from 0 to 6) based on their proportional use of each of topic. More specifically, for each user *u*, we created a numerical topic distribution vector $d_u$:

$$d_u = \left(p_{topic\ 1}, p_{topic\ 2}, \dots, p_{topic\ n}\right)$$

Where *n* is the number of topics and $p_{topic\ i}$ is the proportion of posts user *u* published in topic *i*. These vectors were fed as input to the regression model, with each topic considered as a potential independent variable. To determine a manageable number of topics and facilitate interpretation, we introduced strict thresholds in our stepwise algorithm. The forward selection (inclusion) threshold was set to p-values < 0.003 and the backward selection (exclusion) threshold was set to p-values > 0.005. This step yielded a small set of topics that can be viewed as the most significant predictors of suicide risk.

**Step 4. Analyzing the thematic content of the suicide-related topics.**

In the fourth step, we interpreted the resulting set of topics from the previous step, using three methods: a manual inspection of posts sampled from the topics by three of the authors, a 'consultation' with the currently popular LLM of ChatGPT[7] (which was asked to propose a shared topic for each list of posts, see Table S1), and a well-established analysis named TF-IDF (Term Frequency – Inverse Document Frequency).[8] TF-IDF provides statistical measurements to weight words according to their relevance within each document (or 'topic,' in this case), by taking into consideration both the frequencies of words in the topic and their overall prevalence in the other

topics. This method allowed us to identify the most important words of each topic and thereby to reveal its main theme (Table S2).

The convergence of evidence obtained through these three methods for analyzing thematic content led us to assign a general conceptual label to each topic. Note that we could not provide real examples of postings that were classified into the various topics due to privacy considerations.

Table S1.

*A chatGPT description of the posts in each of the significant buttom-up topics*[7]

| **Topic** | **ChatGPT topic decription** |
|---|---|
| Boredom | The common topic of all the posts you provided is boredom. |
| Wish for something | The topic of the posts appears to be expressing a desire or need for various things or experiences in the authors' lives. These could range from material objects to emotional needs, such as love or support. The specific items or experiences mentioned in the posts vary widely, from tangible items like books and products to abstract concepts like personal growth and emotional connections. |
| View about life | The topic of the following posts is varied, covering a range of subjects and personal thoughts. There isn't a single overarching topic, but rather a collection of individual thoughts, observations, and statements shared by different individuals on social media. These posts touch on subjects like relationships, self-perception, uniqueness, opinions on various matters, personal experiences, and more. |
| Drugs and legalization | The topic of the following posts is primarily about marijuana, including discussions about its legalization, medical benefits, its legal status, and related political and social issues. |

Note. The prompt that was used to receive a response from GPT was: “what is the mutual topic of the following posts: [list of posts that were clustered together in the second step of the research pipeline]”.

Table S2.

*Top 10 TF-IDF words in each of the significant buttom-up topics*

| | Boredom | Wish for something | View about life | Drugs and legalization |
|---|---|---|---|---|
| 1 | Bore (2.85) | Need (0.94) | Matter (0.22) | Marijuana (0.23) |
| 2 | Fuck (0.16) | Life (0.80) | Way (0.20) | Cannabis (0.13) |
| 3 | Im (0.16) | Heart (0.15) | Thing (0.18) | Legal (0.08) |
| 4 | Why (0.13) | Totally (0.12) | Thought (0.17) | Medical (0.06) |
| 5 | Help (0.12) | Important (0.12) | Person (0.13) | Judge (0.05) |
| 6 | Exhausts (0.11) | Repose (0.11) | Quiz (0.12) | Hemp (0.04) |
| 7 | Bulletin (0.10) | Concern (0.09) | Know (0.11) | People (0.03) |
| 8 | Look (0.09) | Adopt (0.09) | Action (0.10) | Weed (0.03) |
| 9 | Emperor (0.09) | Sacrifice (0.08) | Epsiode (0.09) | Plaintiff (0.03) |
| 10 | So (0.09) | Sign (0.08) | One (0.08) | Drug (0.03) |

Note. The words in each column are ordered by their TF-IDF scores (which are presented in parenthesis).

**Step 5. Using a top-down hypothesis testing to further examine the strongest suicide-related topic (i.e., boredom).**

To validate and further examine the results from the previous steps, we re-examined them using more conventional, top-down hypothesis testing methods. Note that the a-priory hypotheses needed to implement this top-down step could have only been formulated after we obtained the results of the previous steps, which suggested that boredom plays a role in the creation or maintenance of the suicide risk (Results).

In this step, as mentioned in the main article, we collected a new sample of 1,062 participants from MTurk and asked them to complete three psychological measurements: (1) the Columbia Suicide Severity Rating Scale (CSSRS),[9,10] (2) the 9-item Patient Health Questionnaire (PHQ-9) measuring depression,[11-13] and (3) the Multidimensional State Boredom Scale (MSBS).[14] This last scale consists of 28 items that aims to assess five theoretical dimensions of boredom. Notably, the central dimension of this scale, the Disengagement dimension that has the largest number of items

and the highest factor loading, reflected the topic of boredom that emerged from the primary Facebook data (see below).

**Similarity analysis**

As a complementary analysis we wished to evaluate the extent to which the bottom-up topic of boredom (which was extracted using the clustering algorithm that was applied to the LLM-based representations of the Facebook posts) is indeed related to the more formal concept of boredom, as might be extracted from the validated questionnaire that measures it. To this end, we used SBERT (see the Methods in the main article) to produce vector representations for the items that comprise the disengagement factor of the Multidimensional State Boredom Scale.[14] In addition, we produced representations of the items of the two other common psychological scales that could serve as control scales for the analysis below – the previously mentioned PHQ-9, which measures depression,[13] and the short version of the well-known Big Five Inventory.[15] In addition to the straightforward comparison with the boredom questionnaire, we were interested in the connection to depression, as the psychological literature draws meaningful connections between boredom and depression (see the introduction section of the main article). Furthermore, we sought to gauge the validity of our findings by employing a broad-spectrum personality assessment, the Big Five questionnaire, as a general measure.

Following this procedure, we computed the similarity score of these scales with the SBERT representation vectors of the postings that were classified to the boredom topic. To calculate this similarity score, we computed the similarity between each sentence in a post, since sentence level is more compatible to match a questionnaire item, and each item in the questionnaire. The final score for each post was obtained by selecting the highest similarity score between a sentence and a questionnaire item. To compute the overall similarity score between the questionnaire and a topic, we took the average of all the similarity scores calculated between the questionnaire and the posts of that topic. When comparing the similarities between the scores of the different scales, we revealed that

the topic of boredom was indeed closer in space to the boredom scale than to the two control scales (Figure S1).

We completed this analysis with a second analysis of 217 random postings that were sampled from the entire Facebook data. We used SBERT to assign representations to these random postings (which matched in their number to the number of postings in the boredom topic) and compared them to the representations of the aforementioned three scales. The random sample was most similar to the Big Five Inventory. We also performed a statistical hypothesis testing, where the null hypothesis is that the similarity between the boredom topic and the random sample to each questionnaire are equal. This comparison revealed that the similarities were significantly different in the cases of the boredom questionnaire ($p < .001$) and the depression questionnaire ($p < .001$) but not in the Big Five Inventory ($p > .05$). It could be concluded then that the topic of boredom reflects, even if not completely, the more formal construct of boredom, as captured by the standard MSBS questionnaire. In other words, this analysis illustrates how LLMs are indeed relevant for the task of identifying hidden psychological constructs.

Our rigorous examination sought to determine the extent to which topic-based boredom posts align with boredom and depression questionnaire items. The results of this analysis present a substantial similarity between boredom-topic posts and items within boredom and depression questionnaires. Nonetheless, further investigation is warranted to comprehensively elucidate the connections and distinctions between topic-based boredom and boredom measured by traditional questionnaires.

Figure S1

*Comparing the representations of the topic of boredom with representations of the items in the boredom scale and in two control scales.*

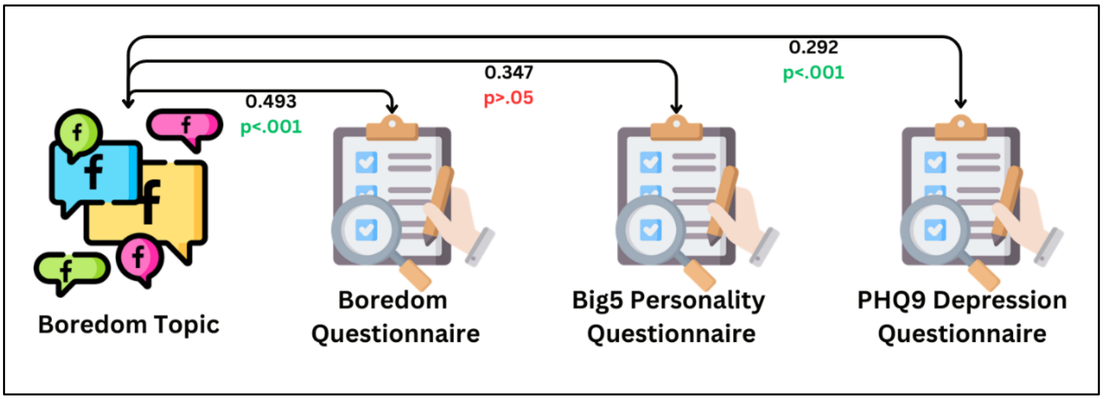


Note. The p-values of this Figure were obtained from a t-test hypothesis testing, comparing the average similarity scores between the topic and the questionnaires to those of randomly selected posts and the questionnaires.

## Path analysis of the Facebook data

The path analysis presented in the main article addressed three psychological constructs: suicide risk, depression, and boredom. The measures used to obtain the first two constructs in the primary Facebook dataset were the same as the measures implemented in the secondary questionnaire dataset (i.e., the CSSRS[16] and the PHQ-9[13]). The third measure of boredom, in contrast, was obtained differently in the two datasets. In the primary Facebook dataset, the boredom scores were assigned to participants based on their usage of the boredom topic, while in the secondary questionnaire dataset the boredom scores were assigned to the participants based on their responses to the boredom scale (i.e., the MSBS).

This difference created a significant gap between the two datasets. While the secondary questionnaire dataset included boredom scores for all the participants (as all were required to complete the boredom questionnaire), in the primary Facebook data, only 3% of the sample (35 of 1,006 participants) had postings that were assigned to the topic of boredom by the clustering algorithm that was used in the study. This low percentage occurred because the clustering algorithm

allowed the assignment of only one topic to each vector representation (Facebook post). In light of this gap, before we implemented the path analysis of the Facebook data, we first enriched and extended the topic of boredom, so more Facebook users could be given boredom scores.

To this end, we created two pools of potentially relevant postings. The first pool consisted of the closest 500 posts to the centroid of the original boredom topic based on the spatial proximity of the vector representations. The reasoning behind this process it that these 500 posts would have been assigned to this cluster with a more tolerant algorithm. The second pool consisted of postings that had explicit boredom-related language (i.e., words with the lingual root of boredom that might have not been classified originally to the boredom topic since the leading topic in these postings required their classification to another topic). We then reviewed the postings of these two pools manually and integrated postings that had clear boredom related content within the original topic of boredom. Illustrative postings of boredom experiences that emerged in this enrichment process are: "I'm mentally drained!", "Why Saturday is so boring?", and "I am bored again, there is no one to talk to". Altogether, we enriched the boredom topic with 53 postings from the first pool and 123 postings from the second pool.

Following this enrichment process, the total number of participants receiving a boredom score larger than zero was 121 (12%), i.e., 86 more participants than the original boredom coding. These enriched boredom scores were then used to compute the path analysis between boredom, depression, and suicide (see the results section of the main article).

## Complete statistics for all path analyses

### Analysis of the primary Facebook data using the Boredom Topic

| Path | coef | se | t | p | Confidence Interval | R^2 |
|---|---|---|---|---|---|---|
| PHQ-9 ~ Boredom Topic | Boredom Topic<br>0.078 | 0.031 | 2.46 | 0.01 | [0.016, 0.139] | 0.006 |
| CSSRS ~ PHQ-9, Boredom Topic | Boredom Topic<br>0.213 | 0.040 | 5.39 | 0.000 | [0.136, 0.291] | |
| | PHQ-9<br>0.597 | 0.040 | 15.08 | 0.000 | [0.519, 0.674] | 0.213 |

| | Effect | SE | p | Confidence Interval |
|---|---|---|---|---|
| Total | 0.260 | 0.043 | 0.000 | [0.174, 0.345] |
| Direct | 0.213 | 0.040 | 0.000 | 0.136, 0.291] |
| Indirect | 0.046 | 0.024 | 0.002 | [0.006, 0.101] |

### Analysis of the secondary, questionnaire data using the Multidimensional State Boredom Scale (MSBS)[14]

| Path | coef | se | t | p | Confidence Interval | R^2 |
|---|---|---|---|---|---|---|
| PHQ-9 ~ MSBS | MSBS<br>0.741 | 0.020 | 35.95 | 0.000 | [0.701, 0.782] | 0.549 |
| CSSRS ~ PHQ-9, MSBS | MSBS<br>-0.080 | 0.048 | -1.68 | 0.093 | [-0.173, 0.013] | |
| | PHQ-9<br>0.687 | 0.048 | 14.43 | 0.000 | [0.593, 0.780] | 0.269 |

| | Effect | SE | p | Confidence Interval |
|---|---|---|---|---|
| Total | 0.429 | 0.035 | 0.000 | [0.360, 0.497] |
| Direct | -0.080 | 0.048 | 0.093 | [-0.173, 0.013] |
| Indirect | 0.508 | 0.051 | 0.000 | [0.412, 0.612] |

## Analysis of the secondary, questionnaire-based dataset using the Boredom Proneness Scale (BPS)[17]

| Path | Coefficient | se | t | p | Confidence Interval | R^2 |
|---|---|---|---|---|---|---|
| PHQ-9 ~ BPS | BPS<br>0.672 | 0.023 | 29.57 | 0.000 | [0.628, 0.717] | 0.452 |
| CSSRS ~ PHQ9, BPS | BPS<br>-0.042 | 0.043 | -0.97 | 0.331 | [-0.127, 0.043] | |
| | PHQ-9<br>0.656 | 0.043 | 15.19 | 0.000 | [0.571, 0.740] | 0.267 |

| | Effect | SE | p | Confidence Interval |
|---|---|---|---|---|
| Total | 0.399 | 0.032 | 0.000 | [0.329, 0.468] |
| Direct | -0.042 | 0.043 | 0.331 | [-0.127, 0.043] |
| Indirect | 0.441 | 0.043 | 0.000 | [0.356, 0.527] |

## Bibliography


1. Ophir Y, Tikochinski R, Asterhan CSC, Sisso I, Reichart R. Deep neural networks detect suicide risk from textual facebook posts. *Scientific Reports*. 2020/10/07 2020;10(1):16685. doi:10.1038/s41598-020-73917-0
2. Reimers N, Gurevych I. Sentence-bert: Sentence embeddings using siamese bert-networks. *arXiv preprint arXiv:190810084*. 2019;
3. Devlin J, Chang M-W, Lee K, Toutanova K. Bert: Pre-training of deep bidirectional transformers for language understanding. *arXiv preprint arXiv:181004805*. 2018;
4. Blei DM, Ng AY, Jordan MI. Latent dirichlet allocation. *the Journal of machine Learning research*. 2003;3:993-1022.
5. McInnes L, Healy J, Melville J. Umap: Uniform manifold approximation and projection for dimension reduction. *arXiv preprint arXiv:180203426*. 2018;
6. Hocking RR. A Biometrics invited paper. The analysis and selection of variables in linear regression. *Biometrics*. 1976:1-49.
7. OpenAI. ChatGPT (September 24 version) [Large language model]. https://chat.openai.com; 2023.
8. Mogotsi IC. Christopher D. Manning, Prabhakar Raghavan, and Hinrich Schütze: Introduction to information retrieval. *Information Retrieval*. 2010/04/01 2010;13(2):192-195. doi:10.1007/s10791-009-9115-y

9. Drapeau CW, Nadorff MR, McCall WV, Titus CE, Barclay N, Payne A. Screening for suicide risk in adult sleep patients. *Sleep Medicine Reviews*. 2019/08/01/ 2019;46:17-26. doi:https://doi.org/10.1016/j.smrv.2019.03.009

10. Weber AN, Michail M, Thompson A, Fiedorowicz JG. Psychiatric emergencies: assessing and managing suicidal ideation. *Medical Clinics*. 2017;101(3):553-571.

11. Spitzer RL, Kroenke K, Williams JBW. Validation and utility of a self-report version of PRIME-MD: the PHQ primary care study. *Jama*. 1999;282(18):1737-1744.

12. El-Den S, Chen TF, Gan Y-L, Wong E, O'Reilly CL. The psychometric properties of depression screening tools in primary healthcare settings: A systematic review. *Journal of Affective Disorders*. 2018/01/01/ 2018;225:503-522. doi:https://doi.org/10.1016/j.jad.2017.08.060

13. Kroenke K, Spitzer RL, Williams JBW. The PHQ-9: Validity of a Brief Depression Severity Measure. *Journal of General Internal Medicine*. 2001;16(9):606-613. doi:10.1046/j.1525-1497.2001.016009606.x

14. Fahlman SA, Mercer-Lynn KB, Flora DB, Eastwood JD. Development and validation of the multidimensional state boredom scale. *Assessment*. 2013;20(1):68-85.

15. Rammstedt B, John OP. Measuring personality in one minute or less: A 10-item short version of the Big Five Inventory in English and German. *Journal of research in Personality*. 2007;41(1):203-212.

16. Posner K, Brown GK, Stanley B, et al. The Columbia–Suicide Severity Rating Scale: initial validity and internal consistency findings from three multisite studies with adolescents and adults. *American Journal of Psychiatry*. 2011;168(12):1266-1277.

17. Farmer R, Sundberg ND. Boredom Proneness--The Development and Correlates of a New Scale. *Journal of Personality Assessment*. 1986/03/01 1986;50(1):4-17. doi:10.1207/s15327752jpa5001_2
